# Nanoparticle size and production efficiency are affected by the presence of fatty acids during albumin nanoparticle fabrication

**DOI:** 10.1371/journal.pone.0189814

**Published:** 2017-12-27

**Authors:** Christian C. Luebbert, Tessa M. Clarke, Roberta Pointet, Grant E. Frahm, Sharon Tam, Barry Lorbetskie, Simon Sauvé, Michael J. W. Johnston

**Affiliations:** Centre for Biologics Evaluation, Biologics and Genetic Therapies Directorate, Health Canada, Ottawa, Ontario, Canada; University of South Australia, AUSTRALIA

## Abstract

We have previously identified extensive glycation, bound fatty acids and increased quantities of protein aggregates in commercially available recombinant HSA (rHSA) expressed in *Oryza sativa* (Asian rice) (OsrHSA) when compared to rHSA from other expression systems. We propose these differences may alter some attributes of nanoparticles fabricated with OsrHSA, as studies have associated greater quantities of aggregates with increased nanoparticle diameters. To determine if this is the case, nanoparticles were fabricated with OsrHSA from various suppliers using ethanol desolvation and subsequent glutaraldehyde cross-linking. All nanoparticles fabricated with OsrHSA showed larger diameters of approximately 20 to 90nm than particles fabricated with either defatted bovine serum albumin (DF-BSA) (100.9 ± 2.8nm) or human plasma albumin (pHSA) (112.0 ± 4.0nm). It was hypothesized that the larger nanoparticle diameters were due to the presence of bound fatty acids and this was confirmed through defatting OsrHSA prior to particle fabrication which yielded particles with diameters similar to those fabricated with pHSA. For additional conformation, DF-BSA was incubated with dodecanoic acid prior to desolvation yielding particles with significantly larger diameters. Further studies showed the increased nanoparticle diameters were due to the bound fatty acids modulating electrostatic interactions between albumin nanoparticles during the desolvation and not changes in protein structure, stability or generation of additional albumin oligomers. Finally the presence of dodecanoic acid was shown to improve doxorubicin loading efficiency onto preformed albumin nanoparticles.

## Introduction

A number of nanoscale drug delivery systems (NDDS) have been approved for clinical treatment of cancer and fungal infections [1–4]. Advantages provided by NDDSs for the delivery of conventional small molecules include passive accumulation at tumor sites due to the fenestrated vasculature leading to the enhanced permeability and retention effect [5–7] and targeting to specific cells using bound targeting ligands [8]. NDDS also reduce the toxicity of an encapsulated drug when compared to the free drug, for example liposomal formulations of doxorubicin show reduced cardiac toxicity, the dose limiting factor for the free drug [9]. Furthermore, NDDS permit the delivery of poorly aqueous-soluble drugs, eliminating the need for specialized excipients. For instance, the conventional delivery of paclitaxel and docetaxel require the use of a Cremophor EL/polysorbate 80 delivery vehicle leading to pharmacological and toxicological issues such as highly variable pharmacokinetics and dose-limiting myelosuppression [10,11]. An albumin-based nanoparticle (Abraxane®) eliminates the need for the delivery vehicle, treatment of the patient with corticosteroids and the use of specialized tubing [12].

Abraxane® or nanoparticle albumin-bound paclitaxel (NAB-paclitaxel) is a 130nm nano particle formulation originally developed by Abraxis BioScience, receiving regulatory approval in from the FDA 2005 and in 2008 from the EMA for the treatment of breast cancer [6,13]. The nanoparticles are formed through high-pressure homogenization of paclitaxel in the presence of serum albumin [14]. Additional reported methods of albumin nanoparticle generation include thermal gelation, self-assembly through modulation of surface hydrophobicity [15] and desolvation used in studies presented here [16].

Delivery of paclitaxel with an albumin nanoparticle has demonstrated improved accumulation of drug at the tumor site as well as higher intratumoral drug concentrations leading to higher portions of patients attaining a pathologic complete response when compared to solvent-based paclitaxel treatment [17]. In addition to the delivery of paclitaxel, NAB technology is also being deployed for the delivery of other drugs such as docetaxol and rapamycin [18].

Two concerns with therapeutics formulated with HSA derived from human plasma (pHSA) are the potential for pathogen contamination and variability in the source material [19], leading to the recent development of recombinant HSA. Studies have been conducted where recombinant HSA (rHSA) replaced pHSA for nanoparticle fabrication. This resulted in dramatic increases in sizes from a size range of 179.9–248.2 nm for pHSA nanoparticles to 296.8 nm for nanoparticles fabricated with rHSA [11]. These researchers attributed the phenomenon to the generation of aggregates during protein purification and freeze drying of their rHSA sample prior to nanoparticle formation [11].

We have extensively studied commercially available rHSA and observed both supplier-to-supplier and lot-to-lot variability in rHSA expressed in *O*. *sativa* (Asian rice) (OsrHSA) when compared to either pHSA or rHSA expressed in yeast, with OsrHSA demonstrating the presence of bound fatty acids, increased arginine/lysine glycation, improved thermal stability and most interestingly the presence of higher molecular weight aggregates [20,21]. With the benefits that a plant based expression system provides in terms of yield and cost effective production [22,23], and the shortages of plasma derived HSA reported in various jurisdictions [24], it is conceivable that rHSA from rice would be used to fabricate nanoparticles for various scientific studies. It is currently unknown how the lot-to-lot and supplier-to-supplier variability we observed could alter the physical attributes of albumin nanoparticles fabricated with OsrHSA. As the presence of protein oligomers, surface charge and charge shielding are major factors in determining nanoparticle size [11,25] we surveyed a number of rHSAs to determine how their altered biophysical properties may affect nanoparticle attributes.

## Materials and methods

### Materials

Essentially fatty acid-free human serum albumin (pHSA, ≥99% purity), recombinant human serum albumin expressed in Saccharomyces cerevisiae (ScrHSA, ≥99% purity, Albucult), human serum albumin expressed in *Pichia pastoris* (PprHSA, ≥99% purity), human serum albumin expressed in *Oryza sativa* (Asian rice) (OsrHSA-Sig, ≥99% purity), and fatty acid-free bovine serum albumin (DF-BSA, ≥99% purity) were purchased from Sigma-Aldrich (St. Louis, MO, USA). Human serum albumin expressed in rice was also obtained from ScienCell Research Laboratories (Carlsbad, CA, USA) (OsrHSA-Sci) (≥99% purity, Oryzogen). Amicon® Ultra 0.5 mL 3000 Da molecular weight cut-off (MWCO) centrifugal filters were obtained from Millipore (Millipore (Canada) Ltd, Etobicoke, ON, Canada). All other chemicals were obtained from Sigma-Aldrich.

### Albumin sample preparation

Albumin samples were prepared as described previously [11,16,26]. Briefly, samples were buffer exchanged into 10 mM NaCl, with Amicon® Ultra 0.5 ml 3000 Da MWCO centrifugal filter devices. Protein concentrations were measured using a BCA assay kit (Sigma-Aldrich) and with NanoDrop protein quantification (Thermo Fisher Scientific Inc., Ottawa, On, Canada). Prior studies have identified the interference of buffers in the desolvation process; thus our experiments utilized 10 mM NaCl as an ionic background for the adjustment with NaOH to achieve a final pH of 8.5 [11,16,26].

### Defatting OsrHSA

OsrHSA-Sig1 (Lot# SLBC7527V) was rapidly defatted using published procedures [21]. Briefly, OsrHSA-Sig1 was exchanged into 5 mM citrate buffer (pH 3.2) as described above in “Albumin sample preparation” to partially unfold the protein to allow for improved removal of bound fatty acids (FA). OsrHSA-Sig1 was applied to a 4 ml column packed with long chain (C13–C18) alkyl ethers-substituted hydroxyalkoxypropyl-dextran (Sigma-Aldrich) equilibrated with citrate buffer (pH 3.2) at a flow rate of 0.5 ml/min run on a GE Healthcare ÄKTApurifier (Piscataway, NJ, USA). The column and all buffer solutions were maintained at room temperature. Protein was eluted from the column with 5 mM citrate buffer (pH 3.2) and the column was regenerated with 10 column volumes of methanol. Defatted OsrHSA-Sig1 (DF-rHSA) was then buffer exchanged into 10mM NaCl and adjusted to pH 8.5 immediately after elution from the column.

### Nanoparticle fabrication

Nanoparticle fabrication was conducted as described by Langer and coworkers [11,26]. Briefly, albumin samples were diluted to 50 mg/ml at a volume of 1ml. To this sample 4ml of ethanol was added at a flow rate of 1 ml/min using a digital PHD ULTRA™ Syringe Pump (Harvard Apparatus, Holliston, MA, USA) while the sample was stirred at 550 rpm. 58.8 μL of glutaraldehyde solution (8% in water) was then added and the sample was stirred for a further 22 hours. The nanoparticles were then centrifuged at 16.1k relative centrifugal force (RCF) for 10 minutes at 4°C with the pellet resuspended with filtered ultrapure water. This washing procedure was repeated two more times prior to a final resuspension in filtered 18Ω water and storage at 4°C. For preparations assessing the effect of fatty acids on nanoparticle properties, DF-BSA was incubated with fatty acids at 37°C for 2 h while stirring, followed by a pH readjustment to pH 8.5 prior to desolvation. Similar methodology has been utilized for loading doxorubicin onto albumin prior to albumin nanoparticle fabrication [16]. Time to desolvation was measured visually as the time point during the four minute ethanol addition at which the solution transitioned from clear to opaque.

### Nanoparticle tracking analysis

A NanoSight NS 300 (Malvern Instruments Ltd, Worcestershire, UK) was utilized for particle sizing and counting of albumin nanoparticles according to the manufacturer’s instructions with appropriate camera levels and flow rates. Reproducibility of counting was determined using 3K-150 Series Particle Counter Standards (152 ± 5 nm, ThermoFisher Scientific, Waltham, Ma, USA). Distribution widths were expressed as:

$$Span=\frac{Dv90-Dv10}{Dv50}$$

Where 90 percent of the distribution lies below the *Dv90* and 10 percent of the population lies below the *Dv10*. The *Dv50* is the median.

### Scanning electron microscopy

Scanning electron microscopy was conducted with a Tescan VegaII XMU SEM (Kohoutovice, Česká republika) according to manufactures instructions. Magnification was set at 50000X.

### Size exclusion chromatography

The size-exclusion chromatography system was a Waters Alliance 2695 Separations Module fitted with a Waters 2996 Photodiode Array Detector (Waters Corporation, Milford, MA, USA). An YMC-Pack Diol-200 column (Product# DL20S05-5008WT, YMC America, Inc., Allentown, PA, USA) with internal dimensions of 500×8.0 mm was used at a flow rate of 0.8 ml/min. The mobile phase consisted of 0.1 M sodium phosphate and 0.15 M sodium chloride (pH 7.0), and peaks were detected at a wavelength of 214 nm. Instrument operation and data acquisition and manipulation were carried out with Waters Empower 2 Chromatography Manager (Waters Corporation) with tabulated results presented as mean +/− standard deviation of 3 separate experiments.

### Circular dichroism spectropolarimetry

Circular dichroism spectropolarimetry (far U/V CD) analysis of albumin samples was conducted as described previously [20,21]. Albumin samples were diluted to 0.15 mg/ml and analyzed in 1 mm quartz cuvettes (Hellma, Müllheim, Germany) with a Jasco 815 spectropolarimeter (Jasco International Co., Ltd. Tokyo, Japan) equipped with a Peltier thermal control unit set to room temperature (22°C), The instrument and thermal control unit were controlled with Spectra Manager Software (Jasco International Co.). Each far U/V CD spectrum for secondary structure analysis represents the average of 5 scans from 260 to 190 nm with a data pitch of 1 nm and a response time of 1 s. Spectra were corrected for buffer. The calculation of secondary structure was conducted using Dichroweb (http://dichroweb.cryst.bbk.ac.uk/html/home.shtml) with the CDSSTR algorithm [27] utilizing reference set 4 (190–240 nm) with results presented as mean +/− standard deviation of 3 separate experiments. Tertiary structural analyses were carried out at 0.45 mg/ml in 10mm quartz cuvettes. Scans covered the near-UV region between 250 and 350 nm with a data pitch of 1nm and a response time of 1s. Spectra were corrected for buffer.

### Doxorubicin loading and release

Doxorubicin was dissolved at 5mg/ml with filtered ultrapure water. Albumin nanoparticles were sized, counted and diluted to 3x10^11^ nanoparticles/ml with filtered ultrapure water, creating a working nanoparticle stock. The working stock was diluted as appropriate with 25 μg of doxorubicin added to each dilution. Loading was conducted over two hours at room temperature with samples mixed through inversion every 15 minutes. Loaded nanoparticles were centrifuged at 16.1k RCF for 30 minutes at 4°C. The supernatant was removed and nanoparticles resuspended in 100mM sodium phosphate buffer, pH 7.4. Unloaded drug was calculated by comparing the doxorubicin in removed supernatant to a doxorubicin standard curve measured at an absorbance of 495nm. Loading efficiency was calculated as:

$$Loading\;Efficiency\;(\%)=\frac{Initial\;Dox\;(\mu g)-Unloaded\;Dox\;(\mu g)}{Initial\;Dox\;(\mu g)}\times 100$$

Due to the differences in nanoparticle size, loading efficiency was expressed as percent of available doxorubicin loaded versus total nanoparticle volume in nm^3^.

To measure doxorubicin release, 3 ml of loaded albumin nanoparticles, containing 75ug of doxorubicin, were placed in 3000 MWCO dialysis bags (SpectrumLabs Inc, Rancho Dominguez, CA, USA) and dialyzed against 40 ml of phosphate buffer while being stirred at 37°C for 4 hours. Released drug was calculated by comparing the doxorubicin in removed dialysate to a doxorubicin standard curve (also incubated at 37°C for 4 hours) measured using a Varian Cary Eclipse Fluorescence Spectrometer controlled with Cary Eclipse Scan Application software (Agilent Technologies, Mississauga, ON, Canada) with an excitation wavelength of 488nm and an emission wavelength of 592 nm [28]. Fluorescence was used to quantify released doxorubicin due to the dilute nature of the released drug. Percentage of drug release was calculated as:

$$Drug\;Release\;(\%)=\frac{(Measured\;Dox\;Released\;(\mu g/ml)\;X\;Total\;Dialysate\;Vol.\;(ml))\;}{Initial\;Dox\;(\mu g)}\times 100$$

### Statistical analysis

Significance was assessed using the Student’s Paired t-test or the Mann-Whitney Rank Sum Test if the assumptions of the *t*-test are not met. SigmaPlot 12.5 software (Systat Software, Inc., San Jose, CA, USA) was used and significance was designated as p < 0.05.

## Results

### Assessment of nanoparticles fabricated from recombinant human serum albumin

Nanoparticles were fabricated using an ethanol desolvation and glutaraldehyde crosslinking method described previously [11,16,26] (Table 1). Nanoparticles fabricated with either defatted bovine serum albumin (DF-BSA) or human serum albumin isolated from human plasma (pHSA) showed diameters similar to those previously reported [25]. We also observed that nanoparticles generated from rHSA, regardless of the expression system or the supplier, were larger than those fabricated from either pHSA or DF-BSA.

**Table 1:** Mean diameters and particle population spans with standard deviations from three separate experiments for albumin nanoparticles generated from albumin from various expression systems/supplier when measured with nanoparticle tracking analysis (n = 3).

Product	Lot #	Mean Diameter (nm)	Span
DF-BSA	021M7401V	101 ± 3	0.31 ± 0.05
	021M7403V	110 ± 4	0.40 ± 0.03
pHSA	SLBD7204V	112 ± 4	0.38 ± 0.09
ScrHSA	SLBM7753V	137 ± 6	0.45± 0.09
OsrHSA-Sig1	SLBP1761V	164 ± 5	0.48 ± 0.07
	SLBN5132V	147 ± 9	0.40 ± 0.05
	SLBJ1196V*	151 ± 11	0.44 ± 0.03
	SLBG7405V*	193 ± 15	0.84 ± 0.24
OsrHSA-Sig2	SLBN8290V	133 ± 7	0.41 ± 0.07
	SLBK8491V	130 ± 3	0.42 ± 0.02
OsrHSA-Sig3	SLBH5563V	164 ± 8	0.50 ± 0.04
OsrHSA-Sci	BJABAA42*	124 ± 3	0.40 ± 0.04
	BJABAA41	162 ± 15	0.65 ± 0.26

*Previous lots assessed by our laboratory

### Fabricating nanoparticles with defatted OsrHSA

OsrHSA-Sig1 (SLBC7527V) was defatted (DF-rHSA) using a long chain (C13-C18) alkyl ethers-substituted hydroxyalkoxypropyl-dextran column. This method was used previously to generate defatted rHSA that demonstrated reduced thermal stability [21]. Nanoparticle fabrication with DF-rHSA reduced particle size (mode) and polydispersity from 187.5 +/- 59.8 nm to 120.1 nm, similar to nanoparticles formulated with pHSA. Nanoparticle fabrication was only undertaken once for DF-rHSA due to low efficiency in the defatting procedure.

### Fabrication of nanoparticles with dodecanoic acid loaded albumin

**Fig 1:**
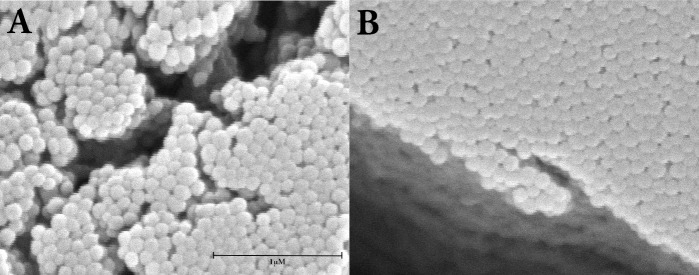
Scanning electron micrographs of albumin nanoparticles. Nanoparticle were fabricated with DF-BSA (Panel A) or dodecanoic acid loaded DF-BSA (6:1 mol:mol, FA:protein) (Panel B). Images are captured at 50000 times magnification Tescan Vega-II XMU SEM. Bar represents 1000nm.

Commercially defatted BSA (DF-BSA) was loaded with dodecanoic acid prior to desolvation and gluterahylde cross-linking. Dodecanoic acid was chosen for these initial experiments due to its solubility when diluted into aqueous albumin solutions and ease of handling. DF-BSA (Lot# 021M7403V) was chosen as it is defatted from the supplier (Sigma-Aldrich). Significantly larger mean and modal diameters were observed for nanoparticles fabricated with dodecanoic acid fatted DF-BSA (6:1 mol:mol, FA:protein) when compared to those free of fatty acids (Mann-Whitney Rank Sum Test, p<0.001, n = 13). No significant changes (*t*-test, p = 0.162, n = 13) in particle population span were noted with dodecanoic acid treatment (Table 2). The increase in albumin nanoparticle diameter due to the presence of dodecanoic acid was orthogonally confirmed with scanning electron microscopy (SEM) (Fig 1). SEM also showed the particles generated with or without dodecanoic acid were spherical in nature. We observed this phenomenon for recombinant albumins as well, for instance loading dodecanoic acid onto OsrHSA-Sci (Lot# BJABAA41) (6:1 mol:mol, FA:protein) increased nanoparticle diameter from 162.3 ± 14.9nm to 249.9 ± 28.2nm.

**Table 2:** Albumin nanoparticle diameter and particle population span with or without incubating dodecanoic acid with DF-BSA prior to desolvation (n = 13).

Product	Condition prior to desolvation	Mean Diameter (nm)	Span
DF-BSA	pH 8.5 2hr 37°C	109 ± 3	0.40 ± 0.03
Lot # 021M7403V	pH 8.5 + dodecanoic acid 2hr 37°C + NaOH (final pH 8.5)	158 ± 13	0.42 ± 0.04

**Fig 2:**
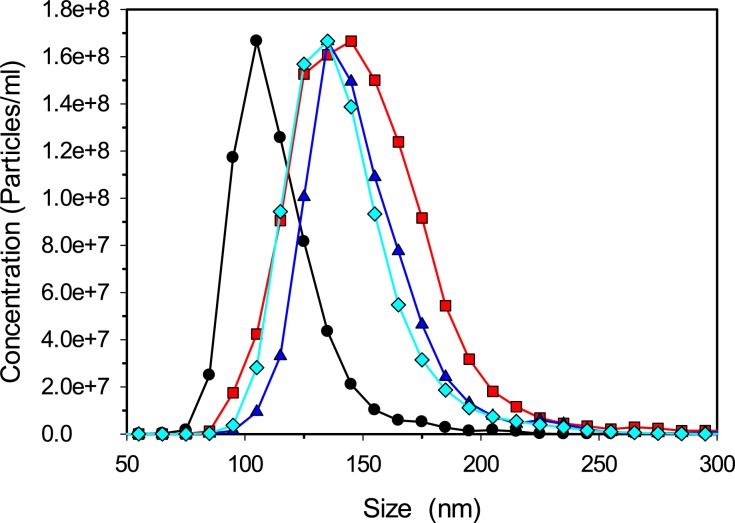
Size distributions for bovine serum albumin nanoparticles. Nanoparticles were fabricated without fatty acids (black circles) or with octanoic acid (red squares), dodecanoic acid (blue triangles) or hexadecanoic acid (cyan diamonds). A NanoSight NS 300 was used to measure particle sizes and data is representative of at least three separate experiments.

Preceding studies have shown that increasing acyl chain length from 8 (octanoic acid) to 18 carbons (stearic acid) increases the binding affinity of saturated fatty acids to albumin [29]. To determine if acyl chain length impacts nanoparticle diameters nanoparticles were fabricated with DF-BSA pre-incubated with octanoic (C8), dodecanoic (C12) or hexadecanoic (C16) acid at a 6:1 fatty acid to protein ratio (mol:mol). Nanoparticles fabricated with the fatty acid-treated BSA all displayed similar increases in diameters to approximately 150 nm (Fig 2).

### Assessment of dodecanoic acid to protein ratio on nanoparticle diameter and fabrication efficiency

**Fig 3:**
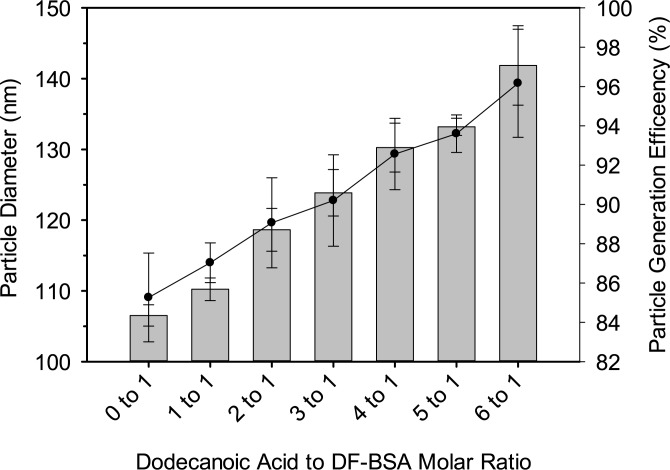
Bovine serum albumin nanoparticle diameters (bar graphs) and particle generation efficiency (solid circles) with increasing dodecanoic acid to protein molar ratios. Each data point represents the mean from three separate experiments with error bars representing the standard deviation.

To further examine the role that fatty acids play in altering the attributes of albumin nanoparticles, increasing molar ratios of dodecanoic acid were incubated with DF-BSA (Lot# 021M7403V) prior to desolvation. A positive correlation was observed between the amount of dodecanoic acid added and particle size. Interestingly, the efficiency of particle generation also showed a positive correlation with increasing molar ratios of dodecanoic acid to DF-BSA (Fig 3).

### Assessment of chloride ions on albumin nanoparticle diameters

Nanoparticle fabrication was undertaken in the presence of excess chloride ions by first lowering the initial pH of DF-BSA prepared at pH 8.5 then lowered to pH 7.75 with HCl (Table 3, Treatment c). This matches the pH shift that results when dodecanoic acid and DF-BSA are incubated at a 6:1 molar ratio (Table 3, Treatment b). After incubation for 2h at 37°C, the pH of each of the above treatments was readjusted to 8.5 prior to desolvation. Both treatment b (the standard dodecanoic acid loading protocol) and treatment c (the corresponding chloride ion control) resulted in increased nanoparticle diameters when compared to DF-BSA nanoparticles fabricated at pH 8.5 with the standard “protein only” protocol (Table 3, Treatment a). Because additional sodium ions (in the form of NaOH) were introduced to treatments b and c during pH readjustment, experiments were conducted to ensure that the larger nanoparticles were not a result of these additional sodium ions. Samples corresponding to treatments b and c, but without final pH readjustment to 8.5 (desolvation therefore occurring at pH 7.75) were made (Table 3, treatments d and e). These preparations produced nanoparticles that were approximately 150 nm in diameter, which were larger than nanoparticles prepared by the standard protein only protocol at pH 7.75 (Table 3, treatment f). Fabrication of DF-BSA nanoparticles at pH 9.5 yielded similar sized particles as those fabricated at pH 8.5 (approximately 108 nm). Taken together, this data suggests that the additional sodium ions present in treatments b and c from pH readjustment are not responsible for the increased nanoparticle diameters of those treatments.

**Table 3:** Comparison of the addition of dodecanoic acid or HCl on albumin nanoparticle diameter(n = 3).

Treatment for DF-BSA (Lot# 021M7403V)	Mean (nm) ± S.D.	Time to desolvation (s) ± S.D.0
a. DF–BSA adjusted to pH 8.5 with NaOH 2hr at 37C	109 ± 0	138 ± 3
b. DF–BSA adjusted to pH 8.5 + dodecanoic acid 2hr 37°C + NaOH (final pH 8.5)	157 ± 2	117 ± 4
c. DF–BSA adjusted to pH 8.5 + HCl 2hr 37°C + NaOH (final pH 8.5)	145 ± 3	124 ± 4
d. DF–BSA adjusted to pH 8.5 + dodecanoic acid 2hr 37°C (final pH 7.75)	164 ± 9	102 ± 6
e. DF–BSA adjusted to pH 8.5 + HCl 2 hr 37°C (final pH 7.75)	158 ± 2	104 ± 2
f. DF-BSA adjusted to pH 7.75 with NaOH 2hr at 37°C	121 ± 1	116 ± 1

### Structure and aggregation of DF-BSA post dodecanoic acid loading

**Fig 4:**
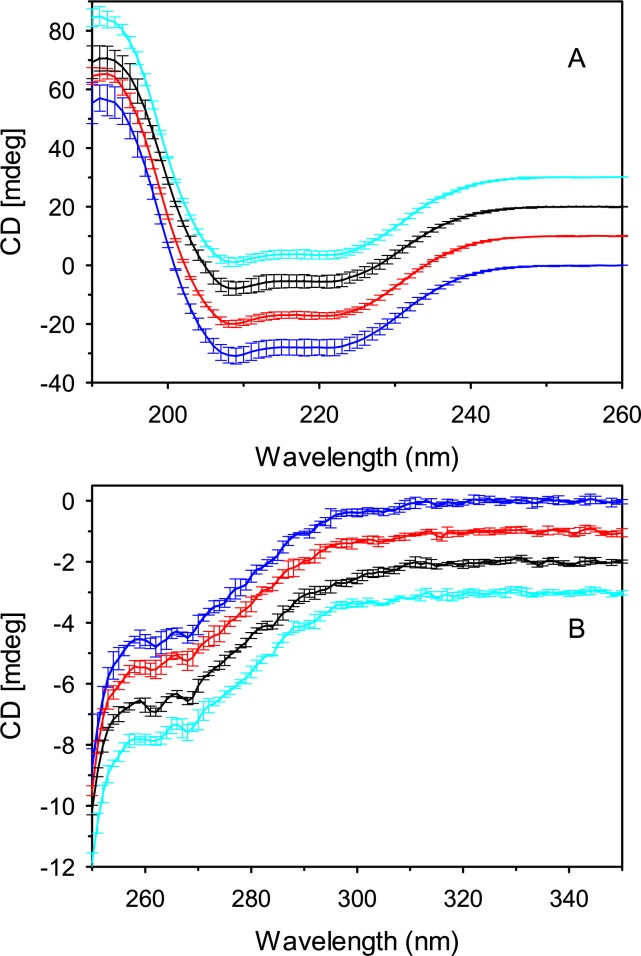
**Far (A) and near (B) UV CD spectra of albumin samples.** DF-BSA pH 8.5 (blue), DF-BSA pH 8.5 incubated 2 hours at 37°C (red), DF-BSA pH 8.5 + dodecanoic acid + NaOH (final pH 8.5) (black) and DF-BSA pH 8.5 + HCL + NaOH (final pH 8.5) (cyan). CD spectra were measured on a Jasco 815 spectropolarimeter. Protein concentrations for all samples were 0.15 mg/ml for far-UV CD and 0.45 mg/ml for near-UV CD. Each spectrum represents the mean from three separate experiments with error bars representing the standard deviation. Spectra were offset 10 mdeg for far UV-CD and 1 mdeg for near UV-CD.

**Fig 5:**
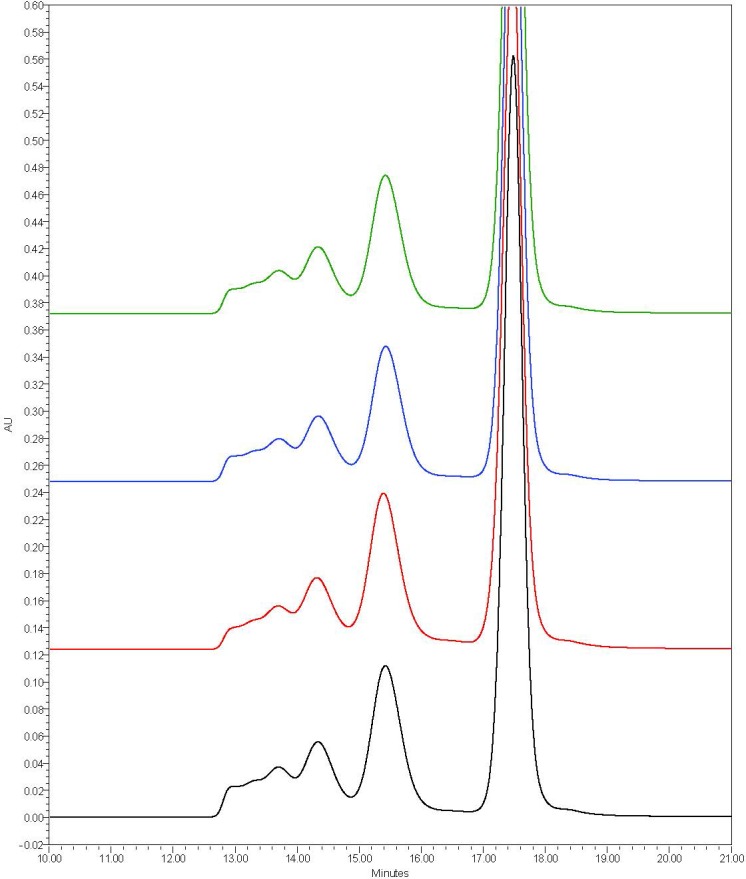
Representative chromatograms of pHSA and rHSA products obtained by SEC analysis. Data was obtained with a YMC-Pack Diol-200 column (500×8.0 mm column) at a flow rate of 0.8 ml/min (0.1 M sodium phosphate, 0.15 M sodium chloride, pH 7.0). Protein elution was monitored at 214 nm. Black: DF-BSA pH 8.5 non-incubated control, Red: DF–BSA adjusted to pH 8.5 with NaOH incubated at 2hr 37°C, Blue: DF–BSA adjusted to pH 8.5 + dodecanoic acid + NaOH (final pH 8.5), Green: DF–BSA adjusted to pH 8.5 + HCl + NaOH (final pH 8.5).

To ascertain if the procedure for loading of dodecanoic acid onto DF-BSA was responsible for the generation of protein aggregates and subsequently the larger nanoparticles, secondary and tertiary structure as well as the aggregate profile of DF-BSA was assessed pre- and post-dodecanoic acid loading. Changes in secondary and tertiary structure were assessed with CD spectropolarimetry and changes in aggregation profile with size exclusion chromatography. Far and near UV CD spectropolarimetry showed no changes to secondary structure (Table 4, Fig 4A) or dramatic alterations to tertiary structure (Fig 4B). Furthermore, examination of the SEC profiles showed a main peak eluting at approximately 17.5 minutes, similar to our previous results [21] (Fig 5). Peaks eluting between 12 and 16 minutes representing DF-BSA dimers and larger aggregates were seen for all treatments with little change in elution time or integrated peak area noted between each treatment (Table 5). Taken together, our data indicates that the increased nanoparticle size observed with DF-BSA treated with dodecanoic acid is not due to structural changes or generation of protein aggregates during the fatty acid loading procedure.

**Table 4:** Mean secondary structure composition (%) and standard deviations for various treatments of DF-BSA (n = 3).

Structure	DF-BSA pH 8.5 –no incubation control	DF–BSA adjusted to pH 8.5 with NaOH, 2 hr 37°C	b. DF–BSA adjusted to pH 8.5 + dodecanoic acid 2 hr 37°C + NaOH (final pH 8.5)	c. DF–BSA adjusted to pH 8.5 + HCl 2 hr 37°C + NaOH (final pH 8.5))
Alpha Helix	64.7 ± 4.0	66.3 ± 2.1	63.7 ± 4.0	61.3 ± 0.6
Beta sheet and turn	15.7 ± 2.5	13.7 ± 1.2	16.7 ± 2.5	17.3 ± 0.6
Unordered	20.3 ± 2.1	20.3 ± 1.5	20.3 ± 2.1	22.0 ± 0.0

**Table 5:** Mean integrated peak areas (percentage of total) and standard deviations from size exclusion chromatography analysis of DF-BSA after various treatment conditions (n = 3).

Peak	DF-BSA pH 8.5 –no incubation control	DF–BSA adjusted to pH 8.5 with NaOH, 2 hr 37°C	b. DF–BSA adjusted to pH 8.5 + dodecanoic acid 2 hr 37°C + NaOH (final pH 8.5)	c. DF–BSA adjusted to pH 8.5 + HCl 2 hr 37°C + NaOH (final pH 8.5)
1	9.75 ± 0.35	7.86 ± 0.27	9.14 ± 0.32	9.00 ± 0.38
2	9.59 ± 0.10	9.55 ± 0.15	9.21 ± 0.15	9.44 ± 0.31
3 (Dimer)	19.90 ± 0.11	22.00 ± 0.34	19.57 ± 0.14	20.06 ± 0.43
4 (Monomer)	60.76 ± 0.43	60.60 ± 0.47	62.08 ± 0.54	61.48 ± 1.05

### Assessment of the stability of unfatted and fatted albumin in increasing concentrations of ethanol

To determine if albumin-bound dodecanoic acid alters protein stability in the presence of increasing ethanol concentrations, far-UV CD analysis of DF-BSA with and without bound dodecanoic acid was carried out. Previous studies have shown that concentrations of ethanol below 50% vol:vol can induce alpha helical structure. At ethanol concentrations greater than 50% (vol:vol) far-UV CD analysis was not possible due to precipitation of the protein. As expected, we observed increasing alpha helical content for DF-BSA without and with bound dodecanoic acid loaded using the standard protocol as ethanol concentration increased to 50% (vol:vol) (Table 6).

**Table 6:** Mean secondary structure composition (%) and standard deviations for DF-BSA with and without bound dodecanoic acid in 10mM NaCl pH 8.5 and varying concentrations of ethanol (vol:vol) (n = 3).

% Ethanol (vol:vol)	Structure (%)	DF-BSA	DF–BSA + Dodecanoic Acid
0% Ethanol	Alpha Helix	64.0 ± 1.0	62.3 ± 2.5
	Beta sheet and turn	15.0 ± 0.0	17.0 ± 1.0
	Unordered	21.3 ± 0.6	21.0 ± 1.0
10% Ethanol	Alpha Helix	67.0 ± 1.7	65.3 ± 5.5
	Beta sheet and turn	13.3 ± 0.6	15.3 ± 3.1
	Unordered	20.0 ± 1.0	19.7 ± 2.5
25% Ethanol	Alpha Helix	70.7 ± 2.1	68.0 ± 1.0
	Beta sheet and turn	12.0 ± 1.0	12.7 ± 0.6
	Unordered	18.3 ± 1.5	19.3 ± 0.6
50% Ethanol	Alpha Helix	70.3 ± 0.6	70.3 ± 3.2
	Beta sheet and turn	12.3 ± 0.6	11.7 ± 1.2
	Unordered	17.3 ± 0.6	18.0 ± 1.7

### Effect of dodecanoic acid on drug loading efficiency and drug retention

**Fig 6:**
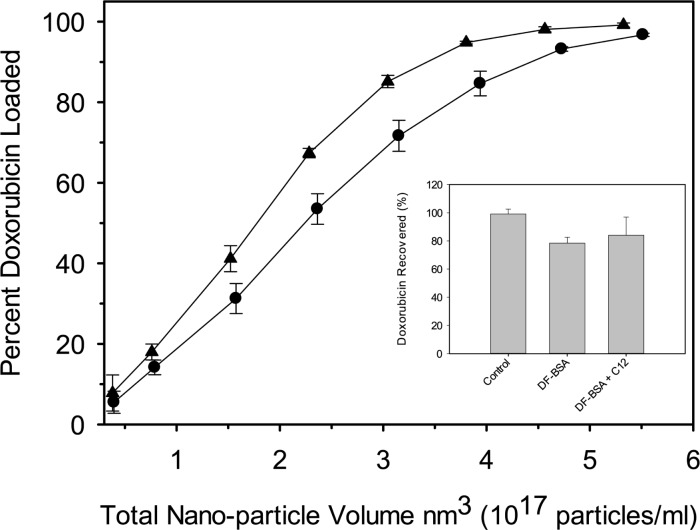
Doxorubicin loading efficiency for DF-BSA nanoparticles fabricated without (circles) and with (triangles) dodecanoic acid normalized for total nanoparticle volume (nm^3^). Each data point represents the mean from five separate experiments with error bars representing the standard deviation. (Inset) *In vitro* doxorubicin release from DF-BSA nanoparticle fabricated without (DF-BSA NPs) and with (DF-BSA C12 NPs) dodecanoic acid. Each data point represents the mean from 3 separate experiments with error bars representing the standard deviation. Dodecanoic acid free nanoparticles for both loading and release studies were fabricated at pH 7.75 to generate particles of similar size to those fabricated with dodecanoic acid.

A constant quantity of doxorubicin (25μg) was loaded into increasing quantities of preformed nanoparticle either fabricated with or without dodecanoic acid. Our data shows that nanoparticles containing dodecanoic acid can more efficiently load doxorubicin when compared to nanoparticles of similar size fabricated without dodecanoic acid (Fig 6). No difference in drug release was observed between doxorubicin-loaded albumin nanoparticles fabricated with and without dodecanoic acid after four hours at 37°C (Fig 6 Inset).

## Discussion

Nanoparticles fabricated in our studies from either defatted bovine serum albumin (DF-BSA) or human serum albumin isolated from human plasma (pHSA) showed diameters similar to those previously reported [25]. We also observed that nanoparticles generated from rHSA, regardless of the expression system or the supplier, were larger than those fabricated from either pHSA or DF-BSA. Langer and coworkers also observed this trend, attributing this to the presence of aggregates generated during protein purification and freeze-drying of rHSA expressed in *P*. *pastoris* [11]. For our studies, all of the albumin samples were used as supplied (lyophilized powders) and underwent minimal sample preparation without further purification or freeze-drying. Additionally, our previous size exclusion chromatography examination of pHSA and various rHSAs showed that OsrHSA-Sig1 (Lot# SLBG7405V) and OsrHSA-Sci (Lot# BJABAA42) had chromatograms with similar dimer/oligomer profiles [21] although here they generated dramatically different sized nanoparticles. The preparation methodology we used and our previous studies suggest that the presence of albumin aggregates is not responsible for the increased nanoparticle size and lot-to-lot variability observed here.

We have noted greater arginine and lysine glycation for OsrHSA [20,21] which could result in changes of localized surface charge on the protein thereby reducing repulsive forces between albumin molecules during desolvation. Studies have shown that albumin surface charge and charge shielding are major determinants of nanoparticle size [16,25] which may explain the increased particle size for nanoparticle fabricated with OsrHSA. This correlation is observed for OsrHSA-Sig1 (Lot#s SLBG7405V and SLBJ1196V) where we earlier observed lot-to-lot differences in the degree and pattern of lysine and arginine glycation [21], with SLBG7405V being more intensely glycated and producing larger nanoparticles compared to SLBJ1196V. However, OsrHSA-Sci (Lot# BJABAA42) has a similar glycation pattern to OrsHSA-Sig1 (Lot# SLBG7405V) [21] but generated smaller particles with less dispersity suggesting that factors other than arginine and lysine glycation are likely responsible for the dramatic differences in particle size and the changes in particle population span.

Finally, we noted that rHSA expressed in rice sourced from Sigma (OsrHSA-Sig1) has exhibited improved thermal stability, as measured by circular dichroism and differential scanning calorimetry [20,21]. For instance OsrHSA-Sig1 (Lot# SLBG7405V) showed a marked improvement in thermal stability over OsrHSA-Sci (Lot# BJABAA42). We have attributed this to the presence of bound fatty acids which are known to stabilize albumin, either by improving thermal stability [30] or by imparting improved resistance to chemical denaturation by either guanidine hydrochloride or urea [31,32]. It is believed that fatty acids provide a linkage between hydrophobic regions and charged amino acid residues [33], thus stabilizing the protein, especially within Domain III [34]. Interestingly, nanoparticles fabricated with this lot of OrsHSA-Sig1 (Lot# SLBG7405V) also demonstrated larger diameters than those fabricated with OsrHSA-Sci (Lot# BJABAA42). Loading of albumin with drugs such as doxorubicin prior to the desolvation process has also been shown to increase nanoparticle size due to potential changes in protein-protein interactions [16]. This suggests the possibility that bound fatty acids on some OsrHSA lots could be responsible for larger diameters of albumin nanoparticles observed when fabricated with these lots.

As well as regulating nanoparticle size, binding specific fatty acids to albumin prior to nanoparticle fabrication could provide therapeutic benefits as N-3 polyunsaturated fatty acids have been shown to affect cell viability, proliferation, and cell cycle progression in breast cancer cell lines [35], as well as decreasing cancer cell line resistance to anticancer therapies [36].

We confirmed that fatty acids were responsible for the increased nanoparticle sizes for some lots of OsrHSA by first defatting an OsrHSA prior to nanoparticle fabrication, which resulted in nanoparticles with diameters similar to those fabricated with DF-BSA. Secondly, we loaded DF-BSA with various fatty acids which resulted in increased nanoparticle diameters regardless of acyl chain length. Although fatting DF-BSA increased nanoparticle diameters, it did not significantly increase nanoparticle population spans, suggesting other factors are responsible for the variable spans noted for the nanoparticles fabricated with rHSA. Previously published studies have suggested low molecular weight impurities not removed during the protein preparation process [11] could be responsible. We believe that further studies should be conducted to determine why some lots of rHSA expressed in rice demonstrate greater levels of polydispersity.

There exist a number of possible mechanisms by which fatty acids increase the diameter of albumin nanoparticles. The first is that the method for loading dodecanoic acid or other fatty acids to DF-BSA could alter the structure or stability of the protein thereby partially unfolding it. Partial protein unfolding has recently been implicated in the generation of protein aggregates [35] the presence of which have been linked to the generation of larger nanoparticles [11]. To ascertain if the procedure for loading dodecanoic acid onto DF-BSA was responsible for the generation of protein aggregates, the larger nanoparticles secondary and tertiary structure as well as the aggregate profile of DF-BSA was assessed pre- and post-dodecanoic acid loading. No changes in secondary or tertiary structure were observed. The aggregation profiles post dodecanoic acid loading had no changed as well, suggesting that the method for loading fatty acids was not responsible for the increased nanoparticle size.

The second potential mechanism involved the improved stability of fatty albumin against thermal and chemical denaturation [20,21,31,34]. Increasing ethanol concentrations have been shown to disrupt native alpha helical structure through the solvation of usually buried hydrophobic side chains and disruption of long-range intramolecular interactions results in non-native alpha helical structures [36]. Bound fatty acids could potentially preserve native secondary structure at higher ethanol concentrations, leading to greater interactions between exposed charged groups on native alpha helical structure. This would lead to reduced protein solubility for fatted DF-BSA and greater protein-protein interaction during desolvation as evidenced by the reduced time to desolvation for preparations containing dodecanoic acid. However, our analysis of secondary structure with far-UV CD showed no differences between DF-BSA and dodecanoic bound DF-BSA up to 50% ethanol (vol:vol) suggesting another mechanism for fatty acid modulation of nanoparticle size.

The third possibility is that bound fatty acid could be affecting albumin nanoparticle diameters through altering protein-protein electrostatic interactions during desolvation. This mechanism is supported by two pieces of evidence. The first is that particle generation efficiency shows a positive correlation with increasing quantities of bound dodecanoic acid. Galisteo-González and Molina-Bolívar observed similar improvements in nanoparticle generation efficiency with increasing ionic strength and attributed this to a reduction of electrostatic repulsive forces due to increased ionic strength shielding surface charges leading to amplified protein-protein interactions during desolvation [25]. Secondly, our data shows that increasing chloride ion concentrations prior to desolvation yields larger diameter nanoparticles in a similar fashion as the binding of dodecanoic acid to DF-BSA.

When fatty anions bind to albumin, the close association of the carboxyl end of the fatty acid with positively charged residues in fatty acids binding sites [29] could alter protein charge (S1 Fig). However, as albumin is negatively charged at pH higher than 5.0, it is difficult for us to reconcile that neutralizing positive residues (thereby making the protein more negative) would lead to greater protein/protein interactions and larger nanoparticles during desolvation. Instead, dodecanoic anions may be screening local regions of positively charged amino acid residues (arginine and lysine) resulting in increased protein-protein interactions through specific surface regions of the protein and, ultimately, the generation of larger nanoparticles during desolvation.

In addition to nanoparticle size, we were curious if the presence of fatty acids altered other physical properties of the nanoparticles such as drug loading. Our data shows that albumin nanoparticles fabricated with dodecanoic acid can more efficiently load doxorubicin when compared to nanoparticles of similar size fabricated without dodecanoic acid. Studies have shown that anthracyclines such as doxorubicin bind strongly to anionic phospholipids through both electrostatic and hydrophobic interactions [37]. The enhanced loading of doxorubicin onto albumin nanoparticles fabricated with dodecanoic acid could be due to the anionic fatty acids providing additional hydrophobic binding sites for the drug on or within the nanoparticle. Alternatively, preceding studies have shown that doxorubicin binds IB subdomain [38] through hydrophilic and hydrophobic interactions [39]. It is possible that dodecanoic acid is preserving doxorubicin binding sites to a greater degree during the desolvation and cross-linking steps of nanoparticle generation.

## Conclusion

In this study we have presented conclusive data, for the first time, that bound fatty acids can alter critical quality attributes of albumin nanoparticles with respect to both nanoparticle size and drug loading efficiency. An expanded understanding of how bound ligands alter albumin nanoparticles generated utilizing desolvation and chemical cross-linking methodology may explain differences in observed albumin nanoparticle diameters between different studies, although similar fabrication techniques were used.

## Supporting information

S1 Fig Recombinant HSA structure with drug binding pockets. Panel 1. Ribbon representation of recombinant human serum albumin from transgenic plant (3SQJ,[24]). The ribbon representation of the human serum albumin structure was colored using the “rainbow” function in Chimera. Panels 2 and 3. Close-ups of Drug Binding Site I, and Drug Binding Site II. Fatty acids (“FA”, myristic acid) near the drug binding sites are shown in sphere representation, and the amino acid side chains that are involved in the binding are shown in sticks representation. Figures were prepared using Chimera ([40]) and the PDB data were retrieved from RCSB data bank ([41]).
(TIF)
