# Supplementary figures and images for: Nanoparticle size and production efficiency are affected by the presence of fatty acids during albumin nanoparticle fabrication
Source: PLoS One. 2017 Dec 27;12(12):e0189814. doi: 10.1371/journal.pone.0189814 (PMC5744977; doi:10.1371/journal.pone.0189814)

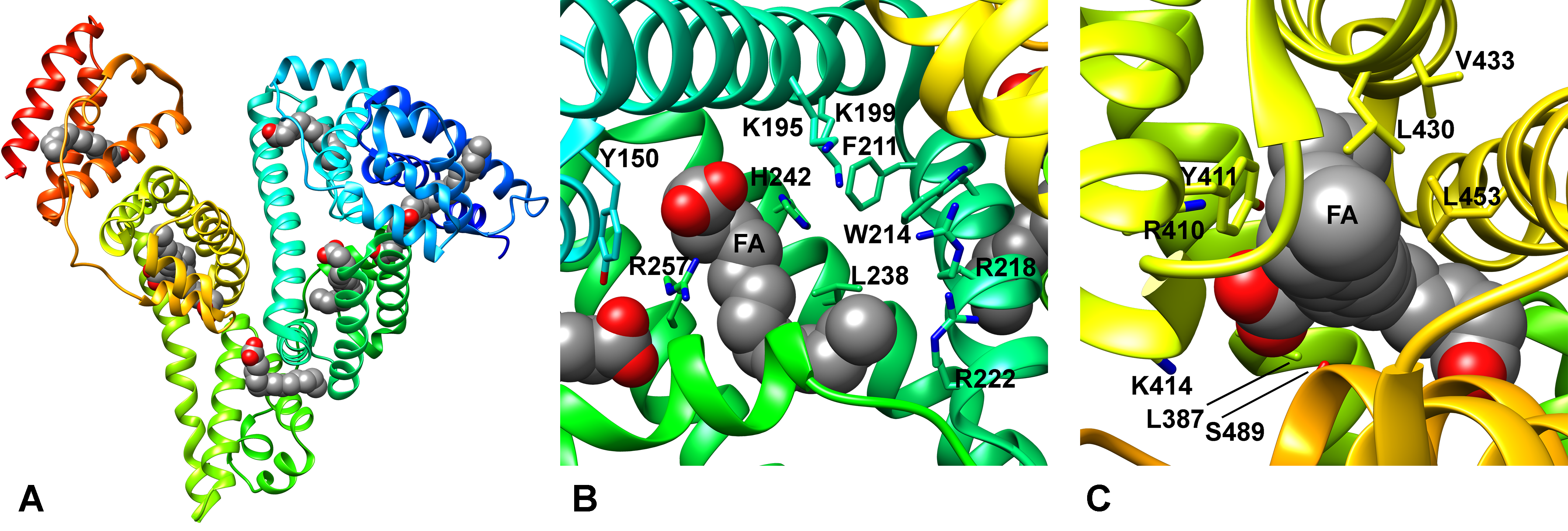

Supplement: S1 Fig — Panel 1. Ribbon representation of recombinant human serum albumin from transgenic plant (3SQJ,[24]). The ribbon representation of the human serum albumin structure was colored using the “rainbow” function in Chimera. Panels 2 and 3. Close-ups of Drug Binding Site I, and Drug Binding Site II. Fatty acids (“FA”, myristic acid) near the drug binding sites are shown in sphere representation, and the amino acid side chains that are involved in the binding are shown in sticks representation. Figures were prepared using Chimera ([40]) and the PDB data were retrieved from RCSB data bank ([41]). (TIF) [file pone.0189814.s001.tif]
